# Qualitative and quantitative analysis methods for quality control of rhubarb in Taiwan’s markets

**DOI:** 10.3389/fphar.2024.1364460

**Published:** 2024-04-30

**Authors:** Thanh-Thuy-Dung Au, Yu-Ling Ho, Yuan-Shiun Chang

**Affiliations:** ^1^ Department of Chinese Pharmaceutical Sciences and Chinese Medicine Resources, College of Chinese Medicine, China Medical University, Taichung, Taiwan; ^2^ Department of Nursing, Hungkuang University, Taichung, Taiwan

**Keywords:** rhubarb, unofficial rhubarb, quality control, anthraquinones, stilbenes

## Abstract

**Introduction:** Rhubarb is a traditional Chinese medicine (TCM) used to release heat and has cathartic effects. Official rhubarb in Taiwan Herbal Pharmacopeias 4th edition (THP 4th) and China Pharmacopeia 2020 (CP 2020) are the roots and rhizomes of *Rheum palmatum* L., *Rheum tanguticum* Maxim. ex Balf., and *Rheum officinale* Baill. However, the *Rheum* genus is a large genus with many different species, and owing to the similarity in appearance and taste with official rhubarb, there needs to be more clarity in the distinction between the species of rhubarb and their applications. Given the time-consuming and complicated extraction and chromatography methods outlined in pharmacopeias, we improved the qualitative analysis and quantitative analysis methods for rhubarb in the market. Hence, we applied our method to identify the species and quality of official and unofficial rhubarb.

**Method:** We analyzed 21 rhubarb samples from the Taiwanese market using a proposed HPLC-based extraction and qualitative analysis employing eight markers: aloe-emodin, rhein, emodin, chrysophanol, physcion, rhapontigenin, rhaponticin, and resveratrol. Additionally, we developed a TLC method for the analysis of rhubarb. KEGG pathway analysis was used to clarify the phytochemical and pharmacological knowledge of official and unofficial rhubarb.

**Results:** Rhein and rhapontigenin emerged as key markers to differentiate official and unofficial rhubarb. Rhapontigenin is abundant in unofficial rhubarb; however, rhein content was low. In contrast, their contents in official rhubarb were opposite to their contents in unofficial rhubarb. The TLC analysis used rhein and rhapontigenin to identify rhubarb in Taiwan’s markets, whereas the KEGG pathway analysis revealed that anthraquinones and stilbenes affected different pathways.

**Discussion:** Eight reference standards were used in this study to propose a quality control method for rhubarb in Taiwanese markets. We propose a rapid extraction method and quantitative analysis of rhubarb to differentiate between official and unofficial rhubarb.

## 1 Introduction

Rhubarb consists of the dried roots and rhizomes of *Rheum palmatum* L., *Rheum tanguticum* Maxim. ex Balf., and *Rheum officinale* Baill according to Taiwan Herbal Pharmacopeia 4th edition (THP 4th) or the underground parts of *R. palmatum* L. and *R. officinale* Baill as per the European Pharmacopoeia 10.0 (EP) (European Pharmacopoeia, 2020; Taiwan Herbal Pharmacopeia, 2024). As a traditional Chinese medicine (TCM), rhubarb was first recorded in Shen-Nong-Ben-Cao-Jing as a laxative, which releases heat and induces catharsis. Rhubarb has been used in numerous decoctions, such as Da-Cheng-Qi-Tang and Xiao-Cheng-Qi-Tang, among others, and in Shang-Han-Lun of Zhang-Zhong-Jing at the end of the Han Dynasty—one of the four classic Chinese medicinal literature works.

Currently, rhubarb is gaining global recognition beyond traditional medicine, with documented pharmacological effects such as bacterial resistance (Wang et al., 2010), anti-inflammatory (Choi et al., 2013), anti-cancer effects (Huang et al., 2007), and enhancement of intestinal barrier function (Xiong et al., 2018).

Rhubarb has been recorded in many countries’ pharmacopeias, including Hong Kong Chinese Materia Medica Standards (Hong Kong Chinese Materia Medica Standards, 2008), THP 4th (Taiwan Herbal Pharmacopeia, 2024), and Pharmacopeia of the People’s Republic of China (Pharmacopoeia of the People’s Republic of China, 2020), with three official species, including *R. palmatum* L., *R. tanguticum* Maxim. Ex Balf., and *R. officinale* Baill. These official rhubarbs contain anthraquinones, notably aloe-emodin, rhein, emodin, chrysophanol, and physcion. Anthraquinones are recognized as the most crucial chemical components in the official rhubarbs with several bioactivities such as laxative (Lombardi et al., 2022), anti-inflammation (Zhang et al., 2020), anti-bacterial (Friedman et al., 2020), anti-diabetic (Arvindekar et al., 2015), and anti-cancer properties (Adnan et al., 2021). Most anthraquinones in TCM or herbs exist in the form of glycosides (Zhao et al., 2014; Jintao et al., 2018) and plant parts like senna leaves (Vilanova-Sanchez et al., 2018), and *Aloe vera* (Hong et al., 2018); such plant parts are used to treat constipation (de Witte, 1993). Free anthraquinones (aglycone anthraquinones) can be absorbed through the small intestine, whereas anthraquinone glycosides reach the large intestine (Feng et al., 2013). Consequently, except for the laxative effect, the active components that traverse the intestinal barrier are free anthraquinone derivatives, rather than anthraquinone glycosides (Fairbairn, 2011; Lin et al., 2017). By contrast, aglycones contain multiple glycosides corresponding to the sugar-binding sites. Therefore, using glycosides as markers is inappropriate because of the limitations in the reference standards for a single high-pressure liquid chromatography photodiode array (HPLC-PDA). Thus, in our study, we focused on quantifying five free anthraquinones, namely, aloe-emodin, emodin, rhein, chrysophanol, and physcion, after hydrolysis, and we optimized their extraction parameters.

In addition, stilbenes are a significant group of chemical components in unofficial rhubarb that are not cataloged in national pharmacopeias. Resveratrol is well known for its strong antioxidant properties (Xia et al., 2017), and it is found in grapes and wine. In addition to resveratrol, rhapontigenin or rhaponticin exhibits anti-inflammatory (Li et al., 2021), cardio-protective (Fan, 2019), anti-hyperlipidemic (Jo et al., 2014), anti-cancer (Kim et al., 2014), and cancer prevention effects (Vervandier-Fasseur and Latruffe, 2019). The most notable stilbene compound in rhubarb is rhaponticin—a glycosidic stilbene not found in official rhubarb (Komatsu et al., 2006; Ye et al., 2007). In EP 10.0, *R. rhaponticum*, an unofficial rhubarb, was examined by thin-layer chromatography (TLC) using rhaponticin as a marker (European Pharmacopoeia, 2020). Thus, rhaponticin serves as an essential stilbene to differentiate official rhubarbs from unofficial ones. Rhapontigenin—aglycone metabolite of rhaponticin (Chen et al., 2020)—exerts extensive anti-allergic (Jo et al., 2014), anti-bacterial (Li et al., 2022), and anti-cancer activities through TGF-β (Yeh et al., 2016). Therefore, rhapontigenin is also an important marker that can be used to distinguish rhubarb from the market.

In recent years, with the discovery of new drugs with pharmacological effects, the identification and quality control of herbal medicines in the market has become increasingly important. *Rheum* is a large genus belonging to the family Polygonaceae, comprising approximately 56 accepted species distributed worldwide (Plants of the World Online, 2023). The genus *Rheum* has many species with similar morphology and taste but comparatively different chemical substances. Moreover, rhubarb in the market is mainly available in a dry form, causing confusion and ambiguity in the distinction between rhubarb species and their applications (Mohtashami et al., 2021). Because it is challenging to be distinguished simply with the naked eyes, adulteration and misrepresentation are prevalent issues.

In Taiwan’s herbal market, most rhubarb is imported from China in the dry form. Through our investigation utilizing TLC with rhaponticin as a marker, we found that most rhubarb in Taiwanese markets is not the official species according to THP 4^th^ standards. Given the limitations of the reference standard, all extraction methods and chromatography in pharmacopeias are based on the hydrolysis principle to detect the aglycones of anthraquinones and stilbenes. These methods are time-consuming and complicated; based on our understanding of the phytochemical properties of rhubarb, we improved qualitative and quantitative analysis methods for rhubarb on the market using TLC and HPLC with eight reference markers, namely, aloe-emodin, rhein, emodin, chrysophanol, physcion, rhaponticin, rhapontigenin (the aglycone of rhaponticin), and resveratrol. This enhanced methodology allows for better clarity regarding the species and quality of both official and unofficial rhubarb.

## 2 Materials and methods

### 2.1 Samples’ preparation and reagents

**FIGURE 1:**
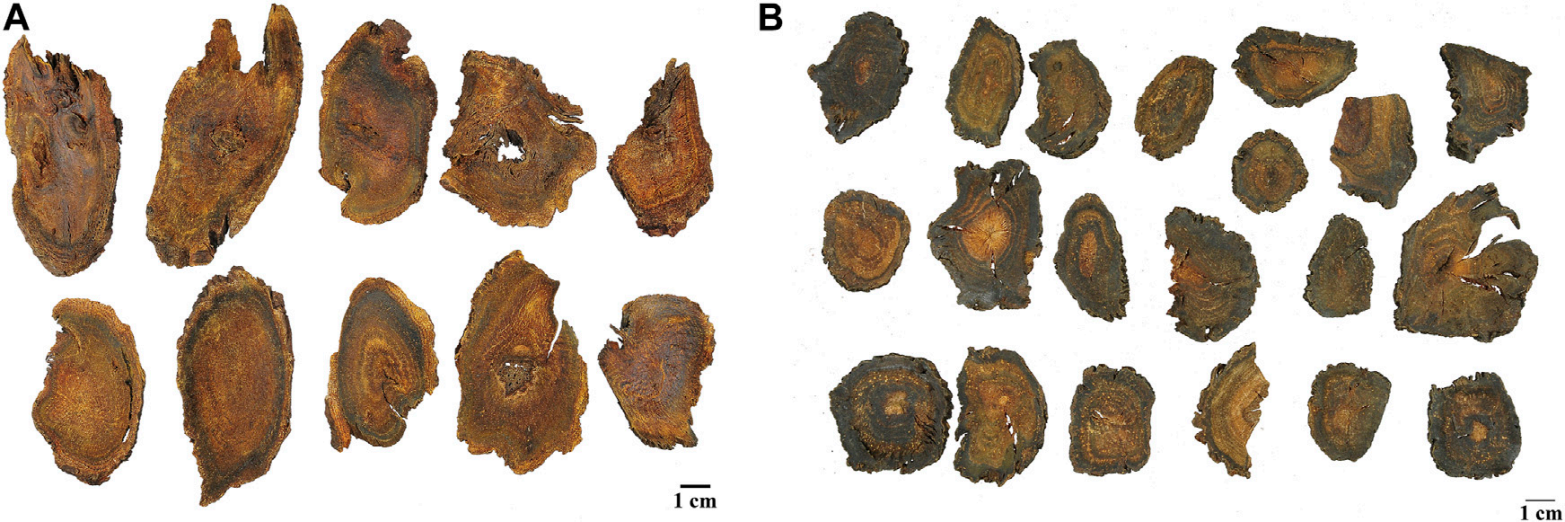
Rhubarb samples from Taiwan market. **(A)** Official rhubarb SP-4 and **(B)** unofficial rhubarb SP-1.

Twenty-one dried samples of rhubarb root and rhizome were collected from different herbal stores in Taiwan and labeled as RH-1 to RH-9 and SP-1 to SP-12. All samples were identified using TLC according to the method described in CP 2020. Samples containing rhaponticin were divided into the unofficial group including RH-5 to RH-8, SP-1, SP-2, SP-3, and SP-5 to SP-12, whereas those that did not contain rhaponticin were classified into the official rhubarb group, including RH-1 to RH-4, RH-9, and SP-4 (Supplementary Figure S1). The photos of samples SP-1 and SP-4, which were used for method validation, are shown in Figure 1. The voucher specimens of all samples were deposited at the Department of Chinese Pharmaceutical Sciences and Chinese Medicine Resources, College of Chinese Medine, China Medical University, Taichung, Taiwan.

Eight reference markers including five anthraquinones and three stilbenes were purchased from different companies as follows: aloe-emodin (purity >95%) and emodin (purity >90%) from Sigma-Aldrich Chemie Gmbh., Taufkirchen, Germany; rhein (purity >98%), chrysophanol (purity >98%), physcion (purity >98%), rhapontigenin (purity >95%), and resveratrol (purity >98%) from Chengdu Purify Phytochemicals Ltd., Chengdu, China; and rhaponticin (purity >98%) from Cayman Chemical Company, Ann Arbor, Michigan, United States of America. Structures of the markers are shown in Supplementary Figure S2. Acetonitrile, methanol, phosphoric acid, hydrochloric acid, acetone, and formic acid were purchased from Merck KGaA, St. Louis, Missouri, United States of America; pentane was procured from ITW Reagents, Monza, Italy; and ethyl acetate was acquired from Honeywell International Inc., Charlotte, North Carolina, United States of America.

### 2.2 High-pressure liquid chromatography

#### 2.2.1 Sample extraction method

Two samples (unofficial SP-1 and official rhubarb SP-4) were used for extraction method validation, including extraction solvent selection, acid concentration selection, extraction duration, and comparison with the extraction method outlined in THP 4th. Accordingly, 0.1 g powder of the sample was extracted in 20 mL of 1%–3% HCl in 70%–100% MeOH, refluxed for 1 h, and filtered. This extraction process was repeated once, the filtrate was combined, and the total volume made up to 100 mL. The resultant mixture was filtered through a 0.45-µm filter, and it was used as the sample solution.

#### 2.2.2 HPLC condition

We tested the method with different columns, including Waters Symmetry C18 column, Xbridge C18 column, Cosmosil C18 column, Dikma C18 column, and Grace Alltima C18 column, all with dimensions 4.6 × 250 mm, 5 μm, under different temperatures, including 25°C, 30°C, and 35°C.

The following conditions were set: solvent A: acetonitrile, solvent B: 0.1% (v/v), 0.05% (v/v), and 0.2% (v/v) phosphoric acid; injection volume: 10 µL; flow rate: 1 mL/min; detector: photodiode array detector (PDA); mobile phase: 0–12 min, 75%–65% solvent B; 12–50 min, 65%–40% solvent B; 50–60 min, 40%–20% solvent B; 60–65 min, 20%–10% solvent B.

### 2.3 Thin-layer chromatography

Six reference standards (rhapontigenin, rhein, aloe-emodin, emodin, chrysophanol, and physcion) were dissolved in methanol to obtain 50 mg/L solution. For the rhubarb extraction method, 0.5 g powder of the sample was extracted in 20 mL of 2% HCl in 70% MeOH, refluxed for 45 min, and filtered to obtain the sample solution (self-development). The development solvent was pentane-ethyl acetate–acetone–formic acid in 15–5–1–0.7 (v/v) (self-development). In the TLC analysis, we used TLC silica gel F254 plate, with 3 µL application as 6 mm bands using a TLC sample semi-automatic applicator Linomat 5 (CAMAG, Muttenz, Switzerland) with Wincat program and Linomat syringes (3 mL; CAMAG, Muttenz, Switzerland). The plate was developed at 80 mm from the lower edge and visualized under 366 nm UV light.

### 2.4 KEGG pathway analysis

For network pharmacology analysis and KEGG analysis, we collected data from different databases as follows: TCMSP for bioactive components, screened the components with OB >20%, DL > 0.1; PubChem and BindingDB for bioactive components—target; UniProt for target—UniProt genes; DisGeNET for UniProt genes—diseases; and KEGG: Kyoto Encyclopedia of Genes and Genomes for genes—pathways.

In this section, we used Cytoscape 3.9.1 and RStudio 4.2.2 software to analyze our results.

### 2.5 Statistical method

To compare the content of eight markers between two groups of samples, we used the *t*-test method using R 4.3.2 and RStudio 4.2.2 to generate boxplots of the data.

## 3 Results

### 3.1 High-pressure liquid chromatography analysis of rhubarb with eight chemical markers

#### 3.1.1 Chemical markers and wavelength absorption of markers

The highest absorption wavelengths of five anthraquinones were closely clustered: aloe-emodin at 225.1 nm, emodin at 221.6 nm, rhein at 229.8 nm, chrysophanol at 223.9 nm, and physcion 222.8 nm. Among the three stilbenes, the highest absorption wavelengths were 325.0, 305.9, and 323.8 nm for rhaponticin, resveratrol, and rhapontigenin, respectively. Thus, we chose 225 and 310 nm wavelengths to observe and measure the anthraquinones and stilbenes, respectively (Supplementary Figure S3).

#### 3.1.2 Sample extraction method

Two samples (unofficial SP-1 and official rhubarb SP-4) were used to validate the extraction method. For extraction solvent selection, we compared the extraction efficiency of various solvent combinations: MeOH, 2% HCl/MeOH, 2% HCl/70% MeOH, 1% HCl/70% MeOH, and 3% HCl/70% MeOH. The results in Supplementary Figure S4 show that an acidic solvent yielded higher peak areas/weights of the eight markers than MeOH. To extract and protect the column better, we selected 2% of HCl over 1% or 3% of HCl as the extraction solvent, partly due to the limitation of the acid hydrolysis method at high temperatures and the high rhaponticin content in the sample hindered complete hydrolysis in unofficial rhubarb with 2% HCl/70% MeOH. However, when compared with the extraction method in THP 4th, satisfactory amounts of free anthraquinones were obtained, whereas all three stilbenes were highly abundant in the unofficial rhubarb SP-1 (Supplementary Figure S4). For extraction times, refluxing for 1 h of reflux and extracting twice was sufficient to extract at least 98% of components (Supplementary Figure S5). Hence, 2% HCl/70% MeOH, reflux of 1 h, and two time extraction were adopted as the extraction methods in this study.

#### 3.1.3 Method validation

After evaluating HPLC condition with five different columns, namely, Waters, Symmetry C18 column (4.6 × 250 mm, 5 µm); Dikma C18 column (4.6 × 250 mm, 5 µm); Cosmosil C18 column (4.6 × 250 mm, 5 µm); Waters, Xbridge C18 column (4.6 × 250 mm, 5 µm); and Grace, Alltima C18 column (4.6 × 250 mm, 5 µm), we chose the Grace, Alltima C18 column, 4.6 × 250 mm, 5 μm, for its optimal separation efficiency and allowable limits of tailing factor for the markers (0.9–1.2). Linearity and calibration studies of rhaponticin, resveratrol, aloe-emodin, rhein, emodin, chrysophanol, and physcion were conducted intraday with a correlation coefficient of *r* ^2^ > 0.999. The details are shown in Supplementary Tables S1, S2.

Relative standard deviation (RSD, %) of precision for the eight markers, rhaponticin, rhapontigenin, resveratrol, aloe-emodin, rhein, emodin, chrysophanol, and physcion, were 0.72%, 0.15%, 0.75%, 0.91%, 0.57%, 0.38%, 0.47%, and 0.66%, respectively. RSD precision was less than 2%. The RSD (%) of the repeatability test for aloe-emodin, rhein, emodin, chrysophanol, and physcion were 3.32%, 2.85%, 2.29%, 4.34%, and 2.70%, respectively, and that of the recovery test were 2.31%, 1.60%, 3.96%, 1.48%, and 1.63%, respectively (Supplementary Table S3). Limits of quantification (LOQ) of rhaponticin, rhapontigenin, resveratrol, aloe-emodin, rhein, emodin, chrysophanol, and physcion were 0.39, 0.97, 0.25, 0.27, 0.29, 0.29, 0.2, and 0.31 mg/L, respectively. Furthermore, limits of detection (LOD) of rhaponticin, rhapontigenin, resveratrol, aloe-emodin, rhein, emodin, chrysophanol, and physcion were 0.2, 0.12, 0.13, 0.15, 0.15, 0.15, 0.1, 0.1, and 0.16 mg/L, respectively (Supplementary Table S3).

#### 3.1.4 Marker content in 13 market rhubarb samples with HPLC analysis

**FIGURE 2:**
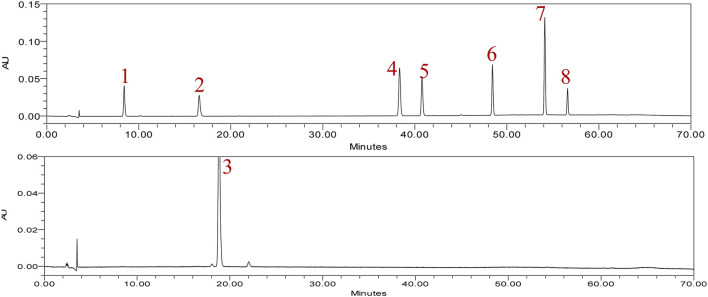
Typical chromatogram of eight reference standards at 225 nm. 1: rhaponticin, 2: resveratrol, 3: rhapontigenin, 4: aloe-emodin, 5: rhein, 6: emodin, 7: chrysophanol, 8: physcion.

**FIGURE 3:**
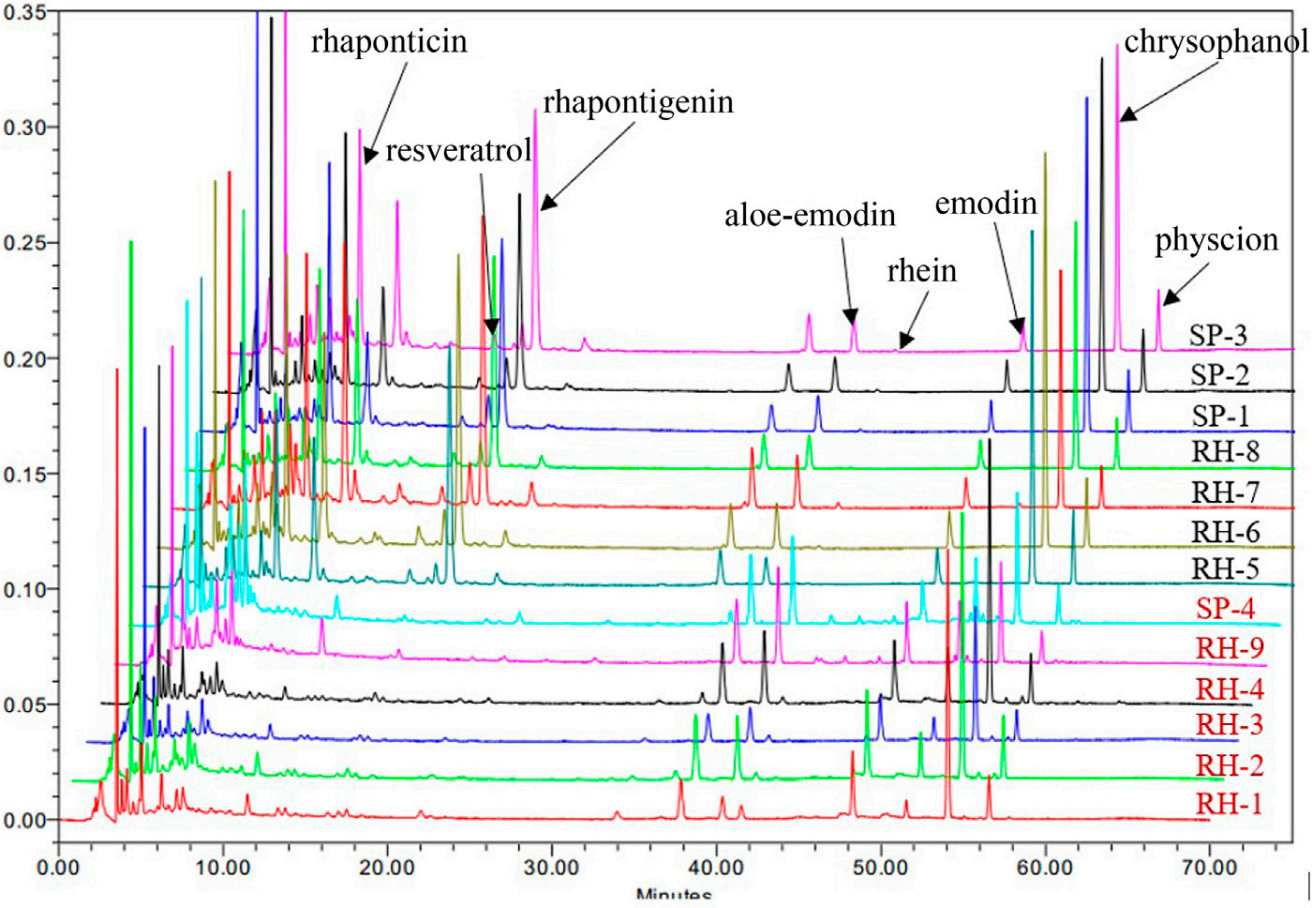
HPLC chromatograms of 13 market rhubarb samples.

In typical chromatography of the reference standards at 225 nm, the retention times of rhaponticin, resveratrol, and rhapontigenin were 8.411, 16.572, and 18.572 min, respectively. Five peaks of aloe-emodin, rhein, emodin, chrysophanol, and physcion appeared at 38.432, 40.747, 48.440, 54.125, and 56.597 min, respectively (Figure 2). The HPLC chromatograms of the 13 market rhubarb samples are depicted in Figure 3.

**FIGURE 4:**
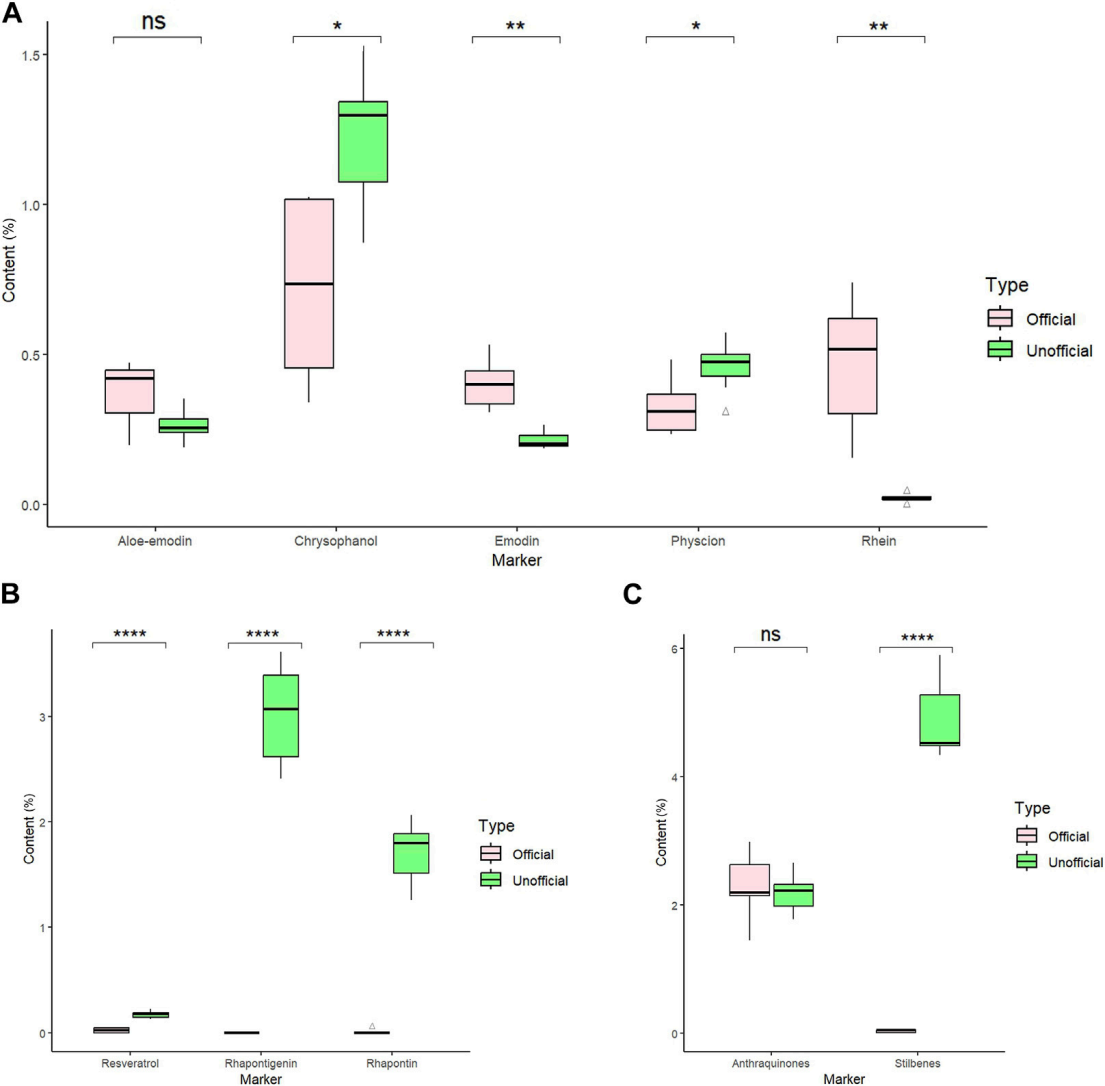
Eight marker content in six official and seven unofficial rhubarb samples. **(A)** Comparison of five anthraquinones content (%) between the two groups of rhubarb. **(B)** Comparison of three stilbenes content (%) between the two groups of rhubarb. **(C)** Comparison of total stilbenes and total anthraquinones content (%) between the two groups. Statistic method: pair *t*-test, ns *p*-value >0.05; *p-value ≦ 0.05; **p-value ≦ 0.01; ***p-value ≦ 0.001; ****p-value ≦ 0.0001.

In the HPLC analysis result, there were 13 rhubarb samples (including seven unofficial rhubarbs and six official rhubarbs); both groups of rhubarb contained anthraquinones: six official rhubarbs contained aloe-emodin, rhein, emodin, chrysophanol, physcion, and no rhapontigenin and rhaponticin were detected; all seven unofficial rhubarb contain aloe-emodin, emodin, chrysophanol, physcion, high content of rhaponticin, and rhapontigenin, but no rhein was detected. Based on our results, the total anthraquinones content in the official rhubarb and unofficial rhubarb groups were 1.45%–2.99% (average 2.28%) and 1.78%–2.66% (average 2.18%), respectively. Thus, the difference in total anthraquinones content was insignificant (*p*-value >0.05). However, there was a very large gap in the content of total stilbenes between two groups of rhubarb, no stilbenes or traces (0%–0.06%, average 0.03%) was detected in the official rhubarb group, whereas a very high total stilbenes (4.33%–5.92%, average 4.90%) was found in the unofficial rhubarb group. The difference in total stilbenes content was significant (*p*-value <0.0001) between two groups of sample. Specifically, in comparing the content of five anthraquinones, chrysophanol and rhein were the two substances with the highest concentration in all samples (RH-9 and SP-4 have the highest rhein content among the five anthraquinones), the content of rhein and emodin in the official rhubarb group were significantly higher than the content of these two substances in the unofficial rhubarb group, but the chrysophanol and physcion content in the official group rhubarb were significantly lower than those in the unofficial rhubarb group. The remaining substance, that is, aloe-emodin, had no significant difference in both groups of the sample (Figure 4). Generally, chrysophanol was the active ingredient with the highest content in all samples (except RH-9 and SP-4), but the differences between unofficial rhubarb and official rhubarb were rhein and rhapontigenin; unofficial rhubarb did not contain rhein or had very low levels of rhein (trace or under LOD), whereas the content of rhapontigenin and rhaponticin in this sample group were very high (Supplementary Table S4). This showed that rhapontigenin and rhein were two active ingredients that played an important role in distinguishing official rhubarb from unofficial rhubarb.

### 3.2 Suggested thin-layer chromatography for qualitative analysis of rhubarb

#### 3.2.1 TLC visualization condition

**FIGURE 5:**
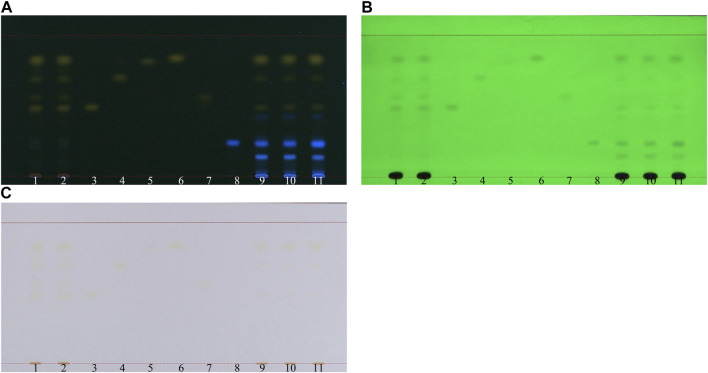
TLC visualization condition for rhubarb: TLC analysis of SP-1 and SP-4 with six reference markers. **(A)** 366 nm UV light, **(B)** 254 nm UV light, and **(C)** white light. 1–2: SP-4, 3: aloe-emodin, 4: emodin, 5: physcion, 6: chrysophanol, 7: rhein, 8: rhapontigenin, 9–10: SP-1, and 11: spike (RH-6 + rhapontigenin).

We visualized TLC under 254 nm UV light, 366 nm UV light, and white light. The results revealed that all six reference standards, namely, aloe-emodin, emodin, physcion, chrysophanol, rhein, and rhapontigenin, were clearly observed under UV light at 366 nm. Therefore, 366 nm was used for visualization in this study (Figure 5).

#### 3.2.2 TLC mobile phase

**FIGURE 6:**
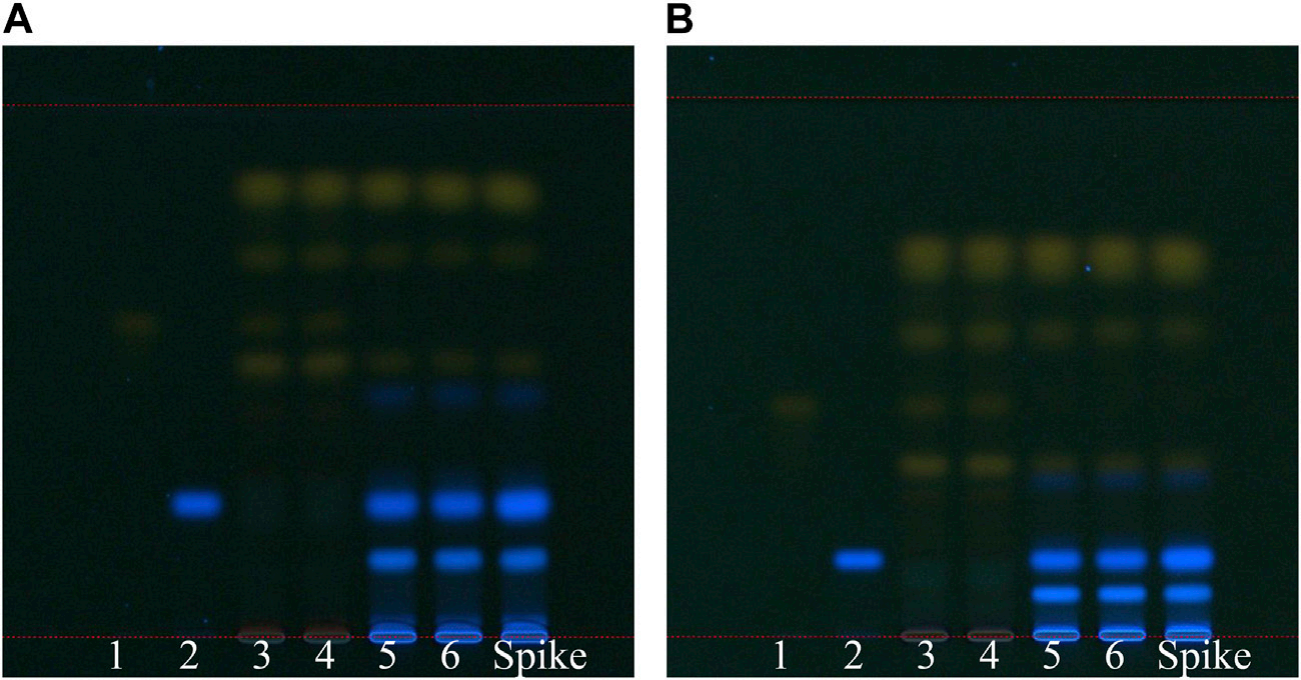
TLC analysis of SP-1 and SP-4 under two development solvents: **(A)** pentane-ethyl acetate–acetone–formic acid 15–5–1–0.7 (v/v) and **(B)** pentane-ethyl acetate–formic acid 15–5–0.5 (v/v). 1: rhein, 2: rhapontigenin, 3–4: SP-4, 5–6: SP-1, 7: spike (SP-1 + rhapontigenin).

We compared TLC efficiency with two solvent systems: pentane-ethyl acetate–acetone–formic acid (system 1) at 15–5–1–0.7 (v/v) and pentane-ethyl acetate–formic acid (system 2) at 15–5–0.5 (v/v). In both solvent systems, we observed yellow bands: from top to bottom, chrysophanol was closely followed by physcion, emodin, rhein, and aloe-emodin. The main fluorescent band was rhapontigenin. Both solvent systems yielded good separation efficiency; however, higher the level, more the chrysophanol and physcion bands tended to overlap. However, according to the HPLC results, chrysophanol and physcion were present in high concentrations in both groups of rhubarb; therefore, the separation of these two substances was rendered unnecessary to distinguish unofficial rhubarb. By contrast, system 1 provided better separation efficiency for rhein and rhapontigenin. Hence, we chose the solvent pentane-ethyl acetate–acetone–formic acid at 15–5–1–0.7 (v/v) for the TLC analysis (Figure 6). In the TLC analysis with this solvent, the retention factor (R*f*) values for rhapontigenin, aloe-emodin, rhein, emodin, physcion, and chrysophanol were 0.24, 0.49, 0.56, 0.70, 0.82, and 0.85, respectively. Because of humidity and air temperature fluctuations, R*f* may have changed slightly, but the difference was not more than 0.02, and the order of the markers was also fixed in all TLC plates for the 21 rhubarb samples.

#### 3.2.3 TLC analysis of 21 rhubarb samples using six reference markers

**FIGURE 7:**
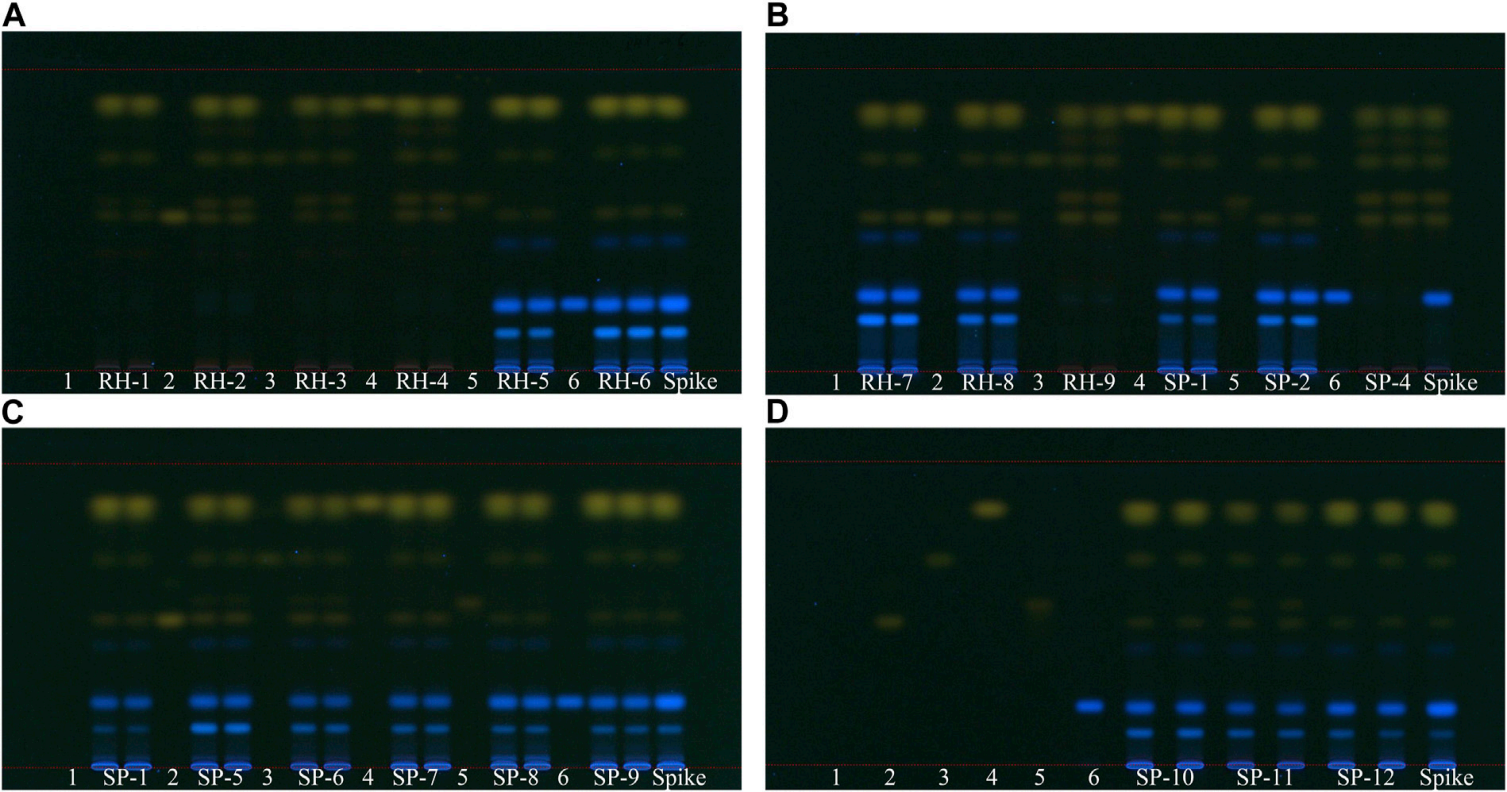
TLC analysis of 21 market rhubarb samples under 366 nm UV light, development solvent A pentane-ethyl acetate–acetone–formic acid 15–5–1–0.7 (v/v). 1: blank (methanol), 2: aloe-emodin, 3: emodin, 4: chrysophanol, 5: rhein, and 6: rhapontigenin. **(A)** Sample RH-1 to RH-6 **(B)** sample RH-7 to RH-9, SP-1, SP-2, and SP-6 **(C)** sample SP-1, SP-5 to SP-9 **(D)** sample SP-10 to SP-12.

In our TLC analysis results, six reference markers, namely, aloe-emodin, emodin, rhein, chrysophanol, physcion, and rhapontigenin, were observed under 366 nm UV light as described earlier. All 21 samples contained aloe-emodin, emodin, chrysophanol, and physcion, whereas samples RH-1, RH-2, RH-3, RH-4, RH-9, and SP- 4 contained rhein but not rhapontigenin. By contrast, unofficial rhubarb samples contained no rhein but rhapontigenin, which was consistent with the HPLC results. Using rhapontigenin as a marker instead of rhaponticin allowed simultaneous TLC with anthraquinones, shortening the experimental process for identifying market rhubarb (Figure 7).

### 3.3 Comparison of stilbenes and anthraquinones using network pharmacology and KEGG analyses

**FIGURE 8:**
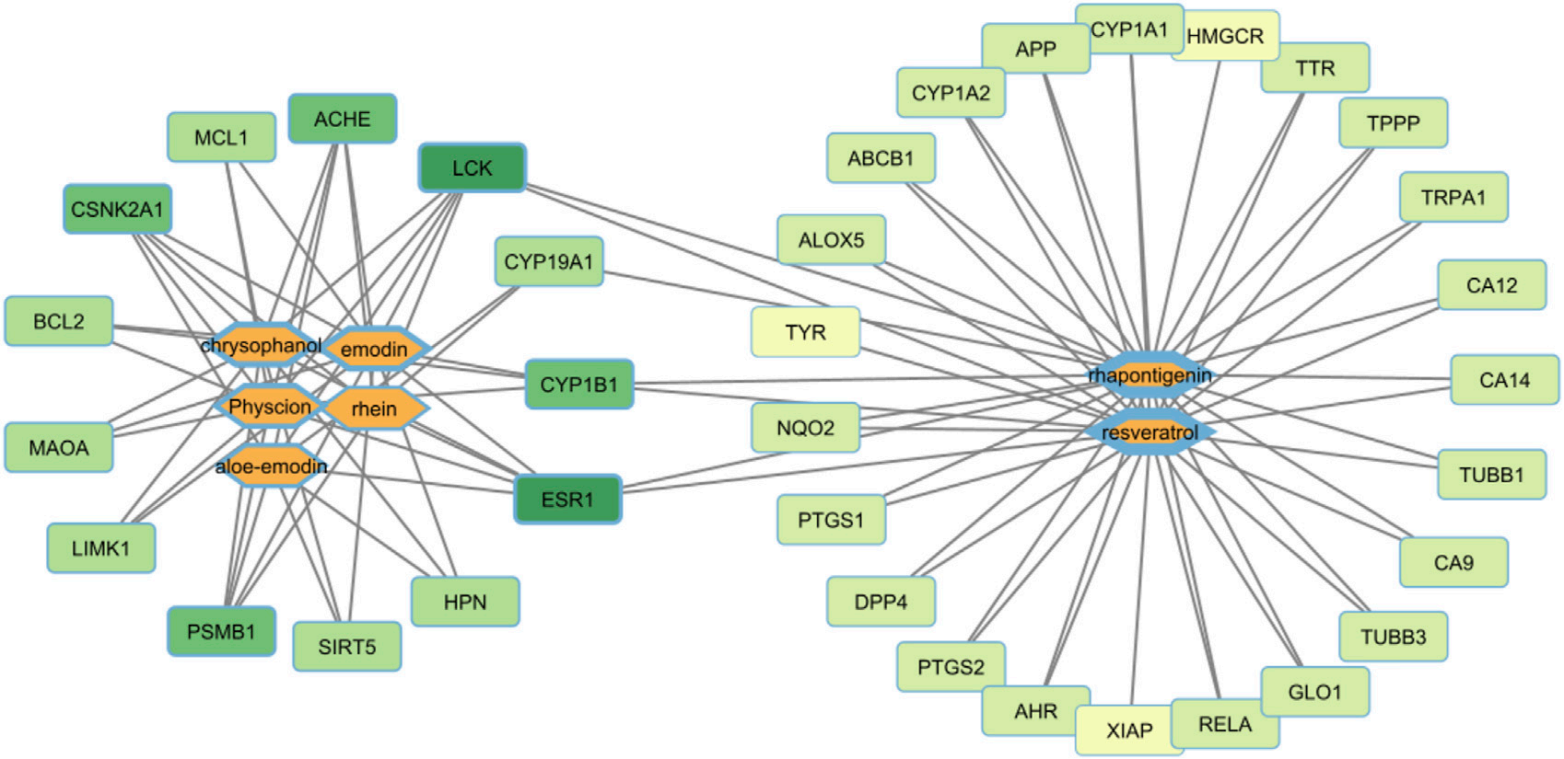
Compound–protein target network of stilbenes and anthraquinones.

Network pharmacology and KEGG analyses revealed that anthraquinones, including aloe-emodin, emodin, rhein, chrysophanol, physcion, and their glycosides, targeted 13 proteins similarly, and stilbenes, including rhapontigenin and resveratrol, targeted 29 proteins. Protein target nomenclature followed that of BindingDB. Both groups of active components had four targets in common. In Figure 8, the target nodes with a darker green color are nodes with a greater degree, indicating that the number of compounds acting on them was higher. The anthraquinone group targets that had the largest degrees were ESR1 (estrogen receptor 1), LCK (human lymphocyte-specific protein tyrosine kinase), and ACHE (acetylcholinesterase). Similarly, for compound nodes, the thicker the border, the more targets that the compound node has. In the anthraquinones group, chrysophanol, physcion, and emodin had the majority of targets. Stilbenes also had a huge target number. Stilbenes had a wide effect, whereas anthraquinones displayed a relatively concentrated effect on a few targets. These results showed that the effects of the two groups were different. The component–target network highlighted the pharmacological differences between stilbenes and anthraquinones (Figure 8).

**FIGURE 9:**
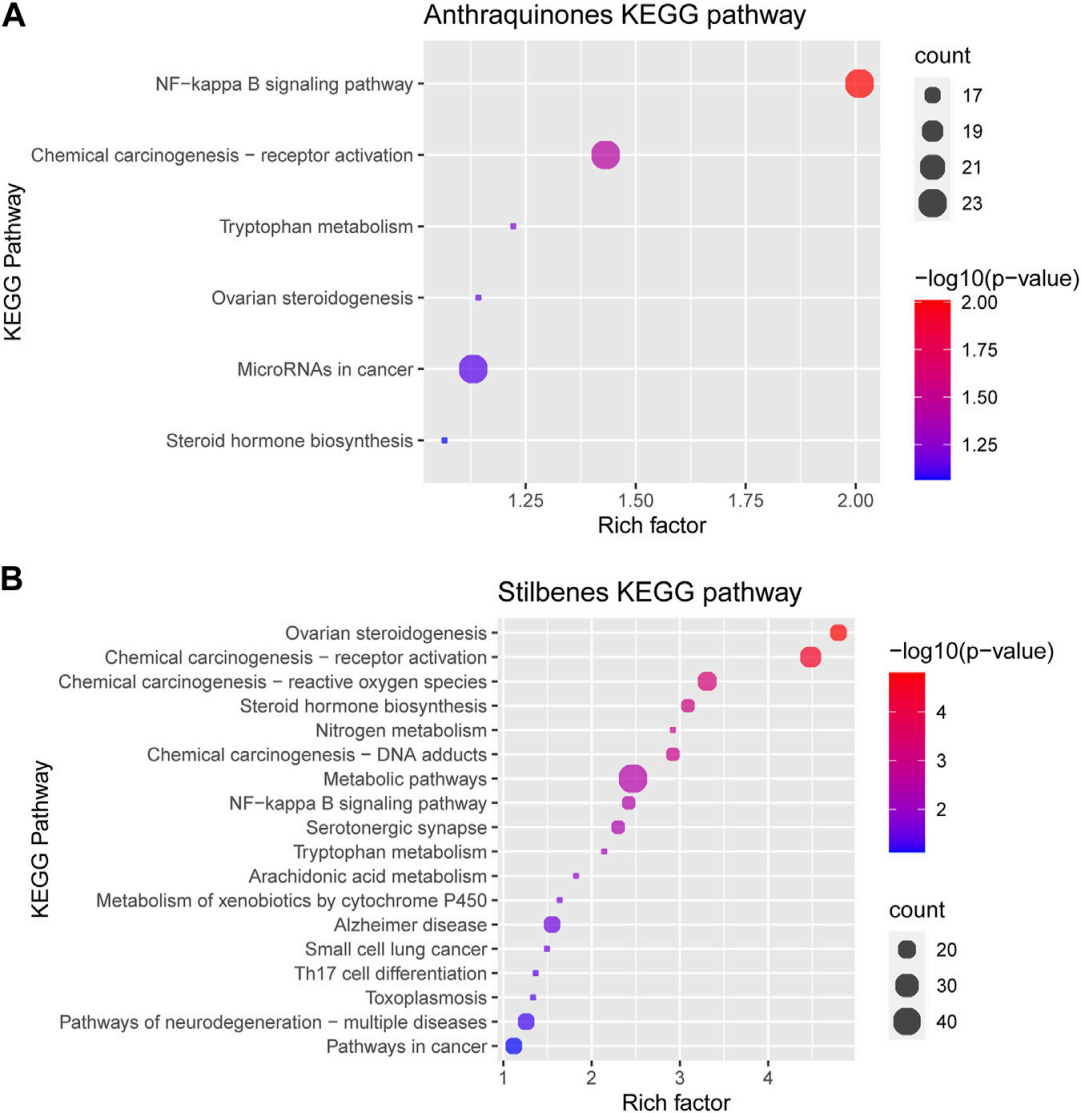
KEGG pathway of **(A)** anthraquinones and **(B)** stilbenes.

From the above network, the KEGG pathway of anthraquinones and stilbenes was conducted to clarify the difference between the two groups of active components. The greater the rich factor, the greater the intensiveness, and the −log10 (*p*-values) represents the level of significance of the pathway. Anthraquinones mainly had anti-inflammatory and anti-cancer effects, whereas stilbenes had ovarian regulatory and anti-cancer effects (Figure 9).

## 4 Discussion

### 4.1 Rhubarb and its adulterant in Taiwan’s market

Many *Rheum* species have already been identified, including *R. rhabarbarum*, *R. emodi*, *R. hotaoense*, and *R. wittrochii* (Wei et al., 2013). Comparing the pharmacological effects of *R. palmatum* and *R. rhabarbarum* L., it was found that *R. rhabarbarum* L. has a stronger small intestinal propulsion-promoting effect but exhibits higher cytotoxicity and acute toxicity in mice (Tianshi et al., 2012). The EP cites *R. rhaponticum* as a species easily confused with the official rhubarb; however, this species is primarily found in Europe and America, whereas *R. rhabarbarum* and others are widely distributed throughout mainland China, Tibet, Malaysia, and other countries. Given the geographical proximity and close cultural history between Taiwan and mainland China, many species, such as *R. rhabarbarum*, *R. emodi*, and *R. australe*, are more likely to surface in the Taiwanese market. In this study, we collected 21 rhubarb samples from the market. Using testing methods based on the pharmacopeia, we discovered that 15 rhubarb samples contained rhaponticin—an active ingredient with anti-bacterial properties, which does not exist in the three official rhubarb varieties listed in the pharmacopeias. Through many previous minor investigations, we found that over 70% of rhubarbs in the market are not official. However, determining the species of these unofficial rhubarbs is difficult because of the diversity of species in genus *Rheum*. Moreover, rhubarb is imported in dried form, making them appear similar, and many genes in the material are damaged during transportation and preservation, rendering gene identification challenging. To date, quantifying eight markers remains the viable method for differentiating unofficial rhubarb in the Taiwanese market. The unofficial rhubarb samples have great similarities, which may be attributed to their homogeneity in the Taiwanese market or that these samples are of the same species. Owing to the economic value of rhubarb in the market, further research is imperative to determine and identify official and unofficial rhubarb.

### 4.2 Qualitative and quantitative analysis methods for rhubarb in Taiwan’s markets

Our research was based on the understanding of market rhubarb, resulting in a quantitative determination method using eight essential markers, including stilbenes and anthraquinones. Although the quantitative HPLC method has yet to be established, changing market conditions necessitates a faster and simpler method with broad applications. According to many pharmacopeias, traditional rhubarb extraction procedure includes reflux, concentration, hydrolysis with HCl, heating, and separation. This procedure provides a cleaner sample with less contamination but eliminates several important active components, especially stilbenes, owing to their high polarity. Eight markers we want to detect are aglycones, and it is necessary to extract them in an acidic solvent at high temperatures to hydrolyze the glycosides. Based on the principles of glycoside hydrolysis, we devised a more straightforward method and validated through HPLC, which demonstrated minimal reduction in anthraquinones content while enabling the detection of stilbenes. This could help in differentiating official rhubarb from unofficial rhubarb.

Additionally, the HPLC conditions used acetonitrile (solvent A) and 0.1% phosphoric acid (solvent B) instead of methanol: 0.1% phosphoric acid. Acetonitrile is considered to have less absorbance, lower pressure requirement, and higher elution capacity than methanol. Therefore, it is generally safe and suitable for HPLC with a UV detector. In addition, the calibration parameters demonstrated the accuracy of our proposed HPLC method.

Based on the results of the HPLC quantitative determination, 13 samples of rhubarb, including six official rhubarb samples and seven unofficial rhubarb samples, showed no significant difference in the total anthraquinones content. Compared with the standard for total anthraquinones in pharmacopeia (NLT 1.5%), only RH-3 was found to be within the limit. However, the anthraquinones content obtained using our extraction method was slightly lower than that obtained using the method described in the THP 4th, so adhering to the extraction method from THP 4th may enable compliance with standards. Notably, all samples exhibited higher concentrations of chrysophanol than other active ingredients, especially in the unofficial rhubarb. Official rhubarb samples contained significant amounts of rhein, whereas the unofficial rhubarb samples contained exceptionally high levels of stilbenes. This result is consistent with the conclusion of a previous study by Xu Jing from China (Jing, 2001), which showed the importance of stilbenes and rhein in distinguishing the two groups of rhubarb. Stilbenes are commonly found in various species, including *R. rhaponticum*, *R. rhabarbarum*, and *R. emodi* (Mohtashami et al., 2021). EP 10.0 indicates rhaponticin as one of the markers for *R. rhaponticum* (European Pharmacopoeia, 2020). In our study, in addition to quantitative HPLC, to compare the two groups of rhubarb, we developed a TLC method using rhapontigenin, the aglycone of rhaponticin with lower polarity, together with five anthraquinones as markers for official and unofficial rhubarb. Based on the TLC results, we successfully differentiated 21 batches into two groups in the rhubarb market. This result demonstrates the applicability of this method to rhubarb in the market.

### 4.3 Comparison of chemical markers in official and unofficial rhubarb through KEGG pathway analysis

Rhubarb contains many active components, in both official and unofficial rhubarb, encompassing anthraquinones, stilbenes, and flavonoids such as myricetin, epicatechin, catechin, epicatechin-3-O-gallate, procyanidin B-2-3,3′-di-O-gallate, procyanidin B1, gallic acid, 1-o-galloyl-b-D-glucose, resveratrol, rhapontigenin, piceatannol, and methoxy resveratrol (Table 1). Anthraquinones is a class of phenolic compounds comprising the 9,10-anthraquinone skeleton and can be found in various species in the *Rheum* genus (Komatsu et al., 2006; Smolarz et al., 2013; Ahmad et al., 2014; Mohtashami et al., 2021). They also exist in many natural herbs, such as *Aloe vera* with main ingredients such as aloin, aloe-emodin, and rhein (Sadiq et al., 2022); *Fallopia multiflora* containing emodin, emodin-8-O-beta-D-glucoside, physcion, and physcion-8-O-beta-D-glucoside (Yan et al., 2010); *Semen cassiae* consisting of eight major anthraquinones (obtusifolin-2-glucoside, aurantio-obtusin, aloe-emodin, rhein, obtusifolin, emodin, chrysophanol, and physcion) (Xu et al., 2012); and *Cassia obtusifolia* L. and *Cassia tora* L. with sennoside A and sennoside B (Franz, 1993; Chen et al., 2023). Anthraquinones include monomers, bianthraquinones, and anthraquinone glycosides, which exhibit several pharmacological effects such as anti-inflammatory (De Oliveira et al., 2021) and anti-obesity properties (Zhang et al., 2018; Lee et al., 2019; Liudvytska and Kolodziejczyk-Czepas, 2022). Most anthraquinones in TCM exist in the form of glycosides (Zhao et al., 2014; Jintao et al., 2018) and the anti-constipation effect is mainly due to the action of anthraquinone glycosides (de Witte, 1993; Le et al., 2021). Sennosides induce the effect of a laxative by boosting intestinal motility, which may be connected with promoting the release of motilin in the intestines, lowering the level of enteric somatostatin, and inhibiting Na+–K + -ATPase activity in the intestinal mucosa (Wu et al., 2020). Free anthraquinones can be absorbed by the small intestine (Feng et al., 2013). As previously mentioned, the efficacy of rhubarb’s active components stems from free anthraquinone derivatives rather than anthraquinone glycosides (Fairbairn, 2011; Lin et al., 2017). Hydroxyl substitution can enhance most of the bioactivities of anthraquinones, such as anti-tumor, anti-pathogenic microorganism activities, antioxidant, and anti-osteoporosis effects (Li and Li, 2018).

**TABLE 1:** Chemical profile of *Rheum* sp.

Material	Chemical profile	References
*R. palmatum*, *R. tanguticum*, and *R. officinale*	Anthraquinones, anthraquinone glucosides, dianthrones, phenylbutanones, stilbenes, flavan-3-ols, procyanidins, galloylglucoses, acylglucoses, gallic acid, and polymeric procyanidins	Komatsu et al. (2006)
*R. palmatum*, *R. tanguticum*, and *R. officinale*	Anthraquinones, anthraquinone glucosides, dianthrones, phenylbutanones, stilbenes, flavan-3-ols, procyanidins, galloylglucoses, acylglucoses, gallic acid, and polymeric procyanidins	Zhang et al. (2015)
*R. palmatum*, *R. tanguticum*, *R. officinale*, *Rumex crispus*, and *R. undulatum*	Sennoside A (3 official species), emodin-glucoside, emodin, chrysophanol (all), and rhaponticin (*R. undulatum*)	Vanmen et al. (2012)
*R. palmatum*, *R. tanguticum*, and *R. officinale*	Tannins, anthrones, anthraquinones, and stilbenes (resveratrol)	Dou et al. (2021)
*R. palmatum*, *R. tanguticum*, *R. officinale*, *R. likoangense*, *R. australe*, and *R. webbianum*	Chinese official rhubarb: anthraquinones (rhein, aloe-emodin, physcion, chrysophanol, and emodin), tannins (catechin), galloyl, gallic acid, caffeic acid, and sennoside. Tibet rhubarb: anthraquinones, stilbenes (resveratrol, rhapontigen, and piceatannol), and tannins (catechin)	Luo et al. (2021)
*Rheum* species *(R. rhaponticum*, *R. rhabarbarum*, *R. emodi*, *R. nobile*, … )	High level of stilbenes	Mohtashami et al. (2021)

KEGG pathway is a database system used to clarify the pharmacological pathways of pharmaceutical substances. To expand our understanding and compare the two groups of rhubarb in the scope of pharmacology in humans, we analyzed the KEGG pathway, in which the rich factor is the % of genes enriched in the total number of genes in the category. According to our result in the KEGG pathway, anthraquinones in rhubarb, including aloe-emodin, emodin, rhein, chrysophanol, and physcion, significantly affected the NF-kappa B signaling pathway—a prototypical pro-inflammatory signaling pathway implicated in chemical carcinogenesis. Emodin, an important anthraquinone with strong anti-inflammatory properties, protects human nucleus pulposus cells against IL-1 beta-induced apoptosis and inflammation *via* inhibiting ROS-mediated activation of NF-kappa B (Zhu et al., 2023). Emodin also suppresses tumor necrosis factor-alpha (TNF-alpha), interleukin-6 (IL-6), iNOS, and COX-2 expression, and inhibits LPS-induced NF-kappa B activation, IKB alpha degradation, phosphorylation of ERK, JNK, and P38 (Yang et al., 2014). In addition, aloe-emodin and its derivatives might be involved in inhibiting Akt, NF-kappa B, and JNK signaling pathways (Shang et al., 2022). Chrysophanol has an anti-inflammatory effect on osteoarthritis in mice *in vitro* by regulating the SIRT6/NF-kappa B and Nrf2/NF-kappa B signaling pathways (Lu et al., 2023). Rhein exerts anti-inflammatory effects by regulating the PPAR-gamma-NF-kappa B-HDAC3 axis (Wen et al., 2020). Physcion glucoside may decrease the expression of pro-inflammatory cytokines and mediators by inhibiting the TGF-beta/NF-kappa B/mitogen-activated protein kinase pathways (Geng et al., 2018).

In addition to anthraquinones, stilbenes can be found in other species of the *Rheum* genus (unofficial rhubarb). Stilbenes, including rhaponticin, resveratrol, and piceatannol, are a class of phenolic compounds with a basic skeleton containing 14 carbons (C6–C2–C6), in which a double-bonded ethylene bridge links the two phenyl moieties (Teka et al., 2022). Stilbenes exhibit several effects like antioxidant (Dou et al., 2017), anti-inflammatory (Tan et al., 2022), and anti-viral properties (Mattio et al., 2020). The three stilbenes discussed in this study were rhaponticin, rhapontigenin, and resveratrol. Resveratrol reduces oxidative stress and improves ovarian function in a rat model (Li and Li, 2018), plays a crucial role in preventing and treating ovarian cancer (Xu et al., 2021), exhibits antioxidant activity with the potential to induce apoptosis, and plays a chemopreventive role in UVR-induced skin carcinogenesis (Aziz and Aziz, 2018). Rhaponticin and desoxyrhaponticin can inhibit fatty acid synthase and are considered potential therapeutic agents to treat cancer (Li et al., 2014). Rhaponticin treatment improves lung cancer *in vitro* (Wang et al., 2021), and it has a protective effect on ovariectomy-induced osteoporosis in rats (Yang et al., 2021).

**FIGURE 10:**
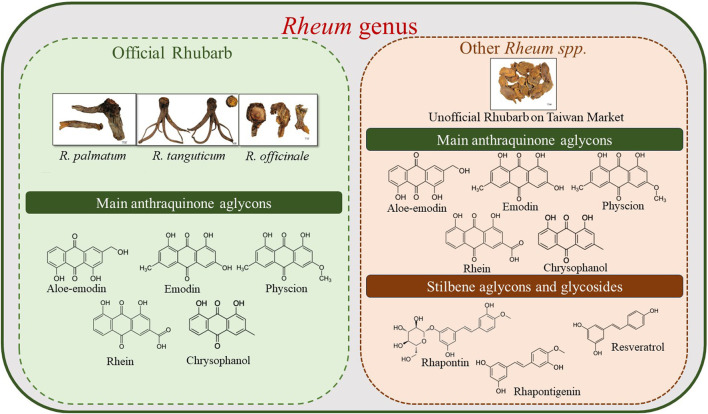
Phytochemical differences between the three *Rheum* species mentioned in Pharmacopeias (*R. palmatum*, *R. tanguticum*, and *R. officinale*) and unofficial rhubarb in Taiwan’s market.

Generally, anthraquinones exhibit a strong anti-inflammatory effect, followed by anti-cancer effects. However, stilbenes have broader spectrum of effects, such as anti-cancer activities related to ovaries and ovarian hormones. The high stilbenes content in unofficial rhubarb indicates its potential value in the Taiwanese market. With more than 50% of rhubarb on the market being unofficial rhubarb, leveraging this resource can maximize the potential of unofficial source of rhubarb. Figure 10 illustrates the phytochemical differences between the three *Rheum* species mentioned in the pharmacopeias (*R*. *palmatum*, *R*. *tanguticum*, and *R*. *officinale*) and other *Rheum* species. This provides a more comprehensive overview of the application of the two groups of rhubarbs and the potential value of unofficial rhubarbs.

### 4.4 The relationship between KEGG pathway analysis and qualitative and quantitative analysis of rhubarb

The relationship between KEGG pathway analysis and qualitative and quantitative analysis can be discussed in several aspects: the presence of active ingredients, the number of active ingredients, the content of active ingredients, and their specific effects.

First, regarding the presence and the number of active ingredients, it can be understood that the herb can act on specific KEGG pathways when there is an active ingredient. When there are many active ingredients, the number of KEGG pathways might differ, and the effect may be more potent. One example is the unofficial rhubarb group, which includes stilbenes and anthraquinones; under examining the KEGG pathway, the unofficial rhubarb group can act on more KEGG pathways than the official rhubarb group, which only contains anthraquinones (Figure 9). However, there is a limitation in determining how strong the effects are. Second, quantitative analysis would provide more information, as many studies have found the drug’s effects are through a dose-dependent pharmacological pathway, with the higher the dose, the stronger the activity. For example, anti-inflammatory research shows that the effect depends on the dose of free anthraquinones (Xiong et al., 2018) or the higher rhaponticin also showed a dose-dependent effect on anti-inflammatory (Zhou et al., 2021). These conclusions suggest that the content of active ingredients and their extraction are also two factors affecting the pharmacological activity of TCM herbs through many pharmacological pathways. Third, through the *p*-value and count index in the KEGG pathway, stilbenes and anthraquinones have strong associations with several pathways, such as anthraquinones with NF-kappa B signaling pathway and stilbenes with ovarian steroidogenesis pathway; thus, with a high concentration of active ingredients, the herb may have some special outstanding medicinal effects rather than the pathway with insignificant *p*-value, such as NF-kappa B signaling pathway and ovarian steroidogenesis pathway.

Qualitative and quantitative analysis methods are used to determine the presence of active ingredients and simultaneously determine the content of that active ingredient in the plant. Meanwhile, the KEGG pathway is a database through which we can know the function and pathway of that active ingredient. Hence, both have a complementary relationship to improve our understanding of active ingredients and drugs.

## 5 Conclusion

Our research analyzed rhubarb in Taiwan’s markets using eight reference standards: official rhubarb contains five anthraquinones, including aloe-emodin, emodin, rhein, chrysophanol, and physcion. In contrast, unofficial rhubarb exhibits high levels of rhapontigenin and anthraquinones, especially chrysophanol, but no rhein was detected. To distinguish between official and unofficial rhubarbs efficiently, we suggested a rapid extraction method and quantitative TLC analysis using rhein and rhapontigenin as pivotal markers. Furthermore, using the KEGG pathway analysis, we elucidated the differences between the pharmacological effects of official and unofficial rhubarbs. This study proposes a quantitative approach to enhance quality control of rhubarb in the market, thereby strengthening our understanding of rhubarb as an alternative.

## Data Availability

The raw data supporting the conclusion of this article will be made available by the authors, without undue reservation.
