# Supplementary material for: Qualitative and quantitative analysis methods for quality control of rhubarb in Taiwan’s markets
Source: Front Pharmacol. 2024 Apr 30;15:1364460. doi: 10.3389/fphar.2024.1364460 (PMC11091417; doi:10.3389/fphar.2024.1364460)
Supplement: Supplementary file 1 [file DataSheet1.pdf]

Qualitative and quantitative analysis method for Rhubarb in Taiwan's markets

Thanh-Thuy-Dung Au[1†], Yu-Ling Ho[2†], Yuan-Shiun Chang[1]

[1]Department of Chinese Pharmaceutical Sciences and Chinese Medicine Resources, College of Chinese Medicine, China Medical University, Taichung, Taiwan.

[2]Department of Nursing, Hungkuang University, Taichung, Taiwan.

[†]These authors have contributed equally to this work and share first authorship

Supplementary

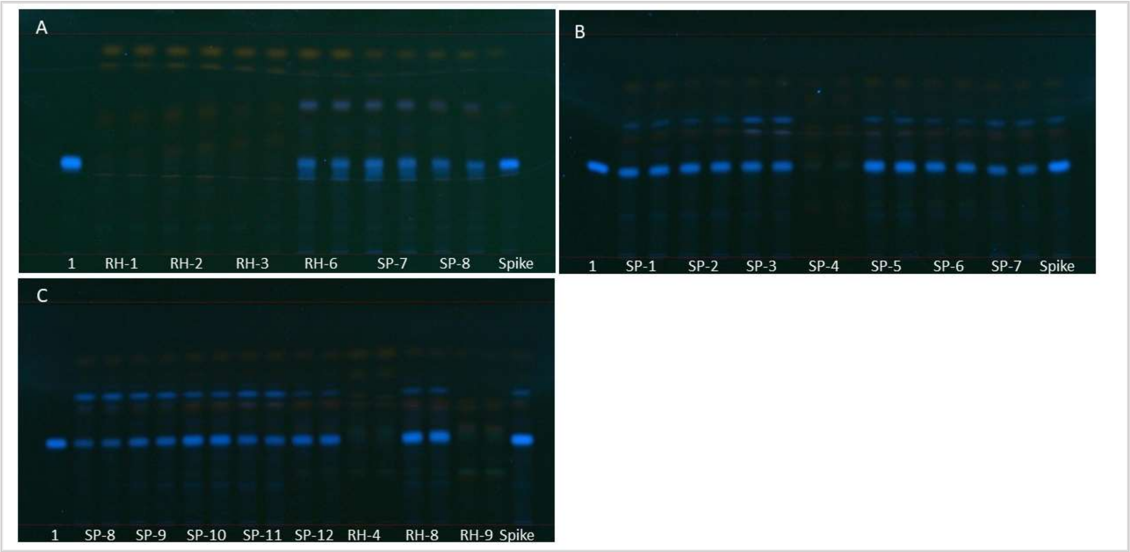

Figure S1 TLC test of rhaponticin according to Pharmacopoeia of the People's Republic of China 2020 (CP2020). **(A)** RH-1 to RH-3 and RH-6 to RH-8 **(B)** SP-1 to SP-7 **(C)** SP-8 to SP-12, RH-4, RH-8, and RH-9. 1: reference standard (rhaponticin/rhapontin), Spike: mixture of sample SP-7 and rhaponticin solution.

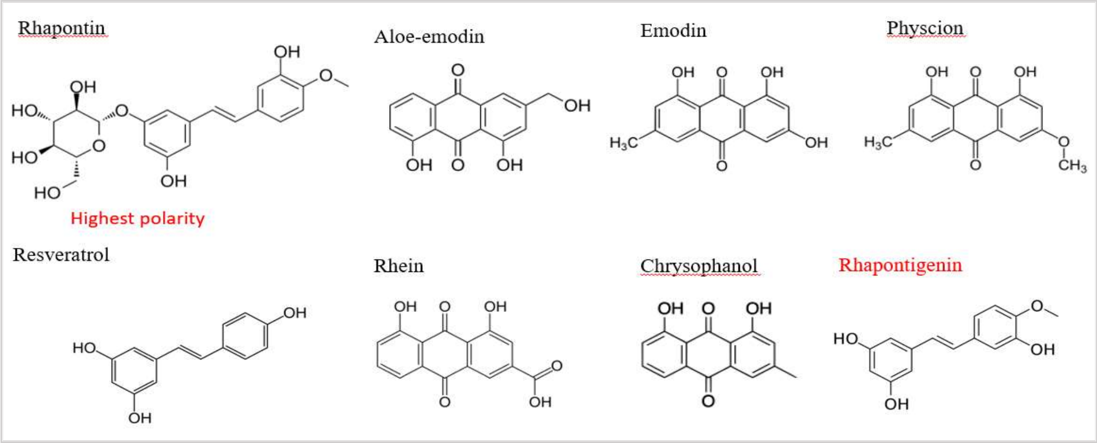

Figure S2 Eight reference markers in the study including five anthraquinones and three stilbenes

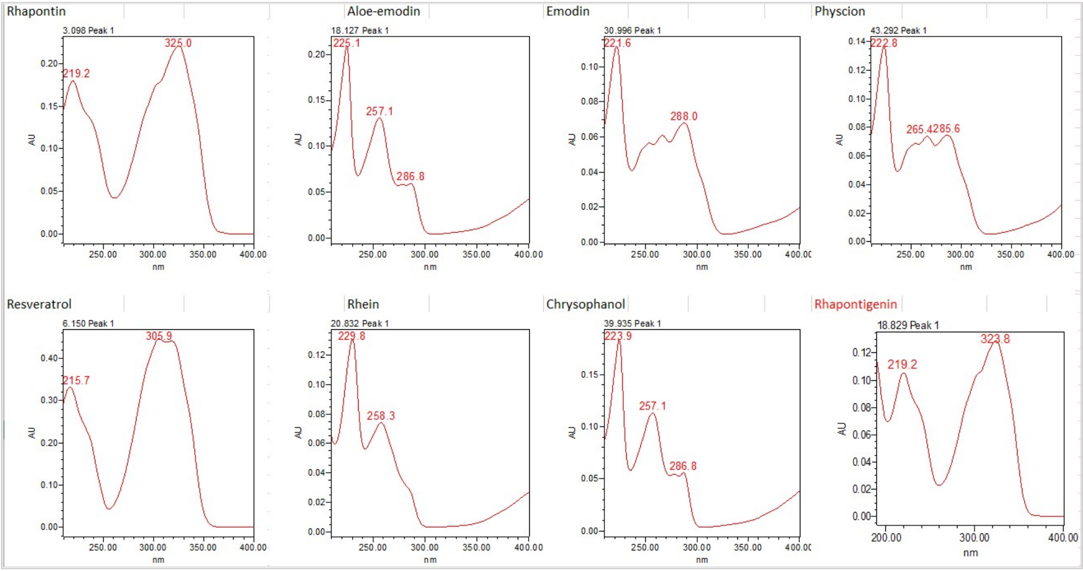

Figure S3 Wavelength absorption of rhaponticin, resveratrol, rhapontigenin, aloe-emodin, emodin, physcion, rhein, and chrysophanol.

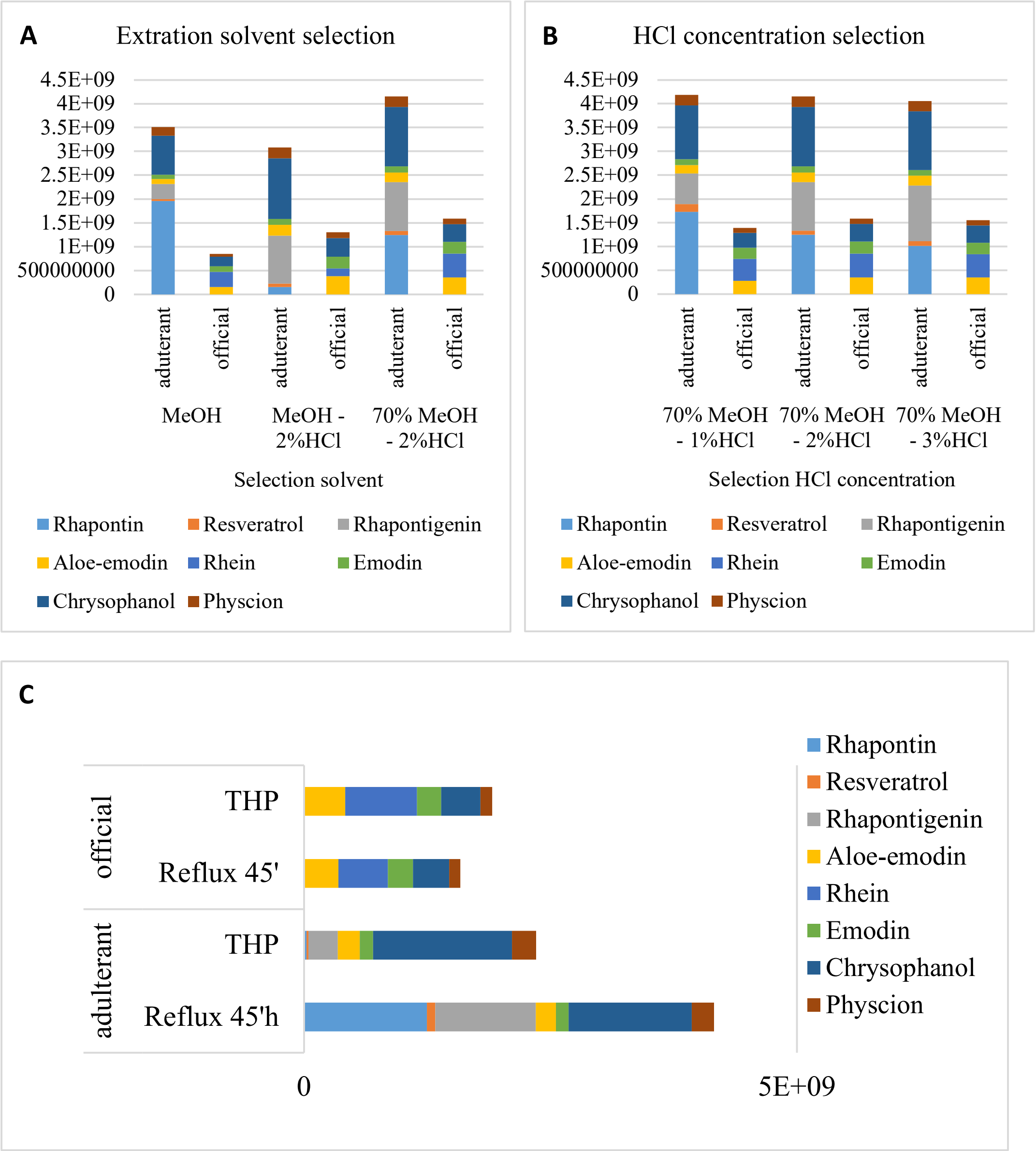

Figure S4 Sample extraction method for HPLC of Rhubarb **(A)** Comparison of different extraction solvents **(B)** Comparison of different HCl concentrations **(C)** Comparison of extraction efficiency by reflux method and THP 4th using 8 reference standards

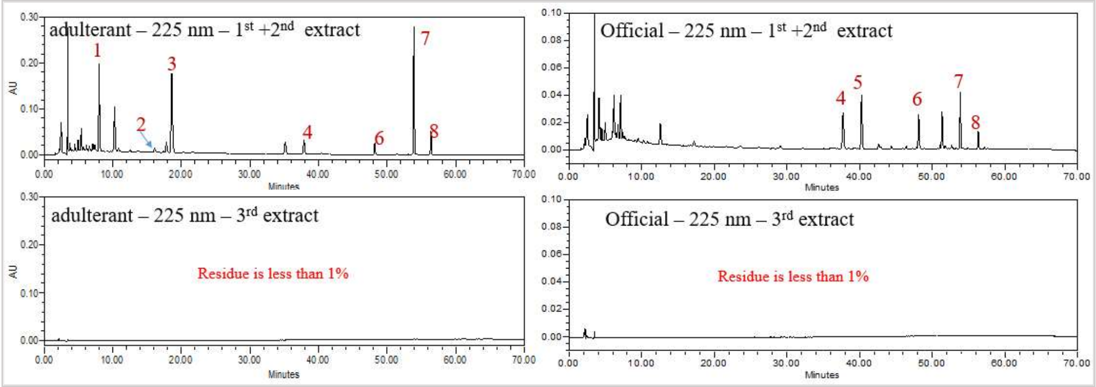

Figure S5 Extraction times of Rhubarb with 45 mins of reflux (sample adulterant: SP-1, sample official: SP-4)

Table S1 Linearity study of 7 markers

Marker	Concentration (mg/L)	Linearity study	Correlation coefficient (r^2)
Rhapontin	0.39-100	y = 36.253x - 8.7345	1.0000
Rhapontigenin	0.12-62	y = 44.554x - 14.727	0.9999
Resveratrol	0.25-65	y = 78.435x - 9.6513	1.0000
Aloe-emodin	0.14-70	y = 80.916x + 3.6966	0.9999
Rhein	0.15-75	y = 60.891x + 7.9131	0.9998
Emodin	0.15-75	y = 63.651x + 2.3463	1.0000
Chrysophanol	0.10-100	y = 89.152x + 20.615	0.9997
Physcion	0.09-22	y = 45.377x + 0.7167	1.0000

Table S2 Calibration curve of 7 markers

Marker	Concentration (mg/L)	Linearity study	Correlation coefficient (r^2)
Rhapontin	6.25-100	y = 36.35x - 15.48	1.0000
Rhapontigenin	3.88-62	y = 44.87x - 28.321	1.0000
Resveratrol	1.02-16.25	y = 76.872x - 4.4758	0.9999
Aloe-emodin	2.19-35.00	y = 82.364x - 7.6244	1.0000
Rhein	4.69-75.00	y = 60.518x + 27.41	0.9999
Emodin	2.34-37.50	y = 63.901x + 3.0623	1.0000
Chrysophanol	6.25-100	y = 88.426x + 71.143	0.9997
Physcion	1.38-22.00	y = 45.44x - 0.2568	1.0000

Table S3 Precision, repeatability test, recovery test, LOD, LOQ of eight reference standards

	Precision (n=5)		Repeatability test (n=5)		Recovery test (n=5)		LOD	LOQ
	Standard Conc. (mg/L)	RSD (%)	Mean (mg/kg)	RSD (%)	Spike amount (mg)	RSD (%)	mg/L	mg/L
Rhapontin	28.50	0.72	-	-	-	-	0.20	0.39
Rhapontigenin	31.00	0.15	-	-	-	-	0.12	0.97
Resveratrol	12.50	0.75	-	-	-	-	0.13	0.25
Aloe-emodin	22.00	0.91	4360.00	3.32	0.43	2.31	0.15	0.27
Rhein	18.75	0.57	7171.15	2.85	0.72	1.6	0.15	0.29
Emodin	17.75	0.38	3741.04	2.29	0.37	3.96	0.15	0.29
Chrysophanol	25.00	0.47	3340.75	4.34	0.33	1.48	0.10	0.20
Physcion	12.50	0.66	2383.90	2.7	0.43	1.63	0.16	0.31

Table S4 Content (%) of eight markers in 13 samples of Rhubarb

Type	ID	Rhapontin (%)	Resveratrol (%)	Rhapontigenin (%)	Aloe-emodin (%)	Rhein (%)	Emodin (%)	Chryso-phanol (%)	Physcion (%)	total	
										Anthraquinone (%)	Stilbenes (%)
Official Rhubarb	**RH-1**	0.00	0.05	0.00	0.27	0.15	0.42	1.02	0.34	2.21	0.05
	**RH-2**	0.00	0.05	0.00	0.45	0.50	0.53	1.03	0.48	2.99	0.05
	**RH-3**	0.00	0.00	0.00	0.20	0.24	0.31	0.47	0.24	1.45	0.00
	**RH-4**	0.00	0.04	0.00	0.40	0.54	0.45	1.00	0.38	2.77	0.04
	**RH-9**	0.06	0.00	0.00	0.47	0.65	0.32	0.45	0.28	2.17	0.06
	**SP-4**	0.00	0.00	0.00	0.44	0.74	0.37	0.34	0.24	2.13	0.00
	**min**	0.00	0.00	0.00	0.20	0.15	0.31	0.34	0.24	**1.45**	**0.00**
	**max**	0.06	0.05	0.00	0.47	0.74	0.53	1.03	0.48	**2.99**	**0.06**
	**average**	0.01	0.02	0.00	0.37	0.47	0.40	0.72	0.32	**2.28**	**0.03**
	**SD**	0.02	0.02	0.00	0.11	0.23	0.08	0.33	0.10	**0.54**	**0.03**
Unofficial Rhubarb	**RH-5**	1.26	0.18	3.07	0.19	0.00	0.25	1.39	0.57	2.39	4.51
	**RH-6**	2.07	0.22	3.63	0.31	0.02	0.27	1.53	0.52	2.66	5.92
	**RH-7**	1.80	0.20	3.64	0.35	0.04	0.20	0.87	0.31	1.78	5.64
	**RH-8**	1.45	0.17	2.72	0.24	0.02	0.20	0.95	0.39	1.79	4.33
	**SP-1**	1.91	0.13	2.41	0.26	0.02	0.19	1.30	0.47	2.23	4.45
	**SP-2**	1.87	0.13	2.53	0.24	0.02	0.19	1.30	0.48	2.22	4.52
	**SP-3**	1.58	0.17	3.16	0.26	0.02	0.21	1.20	0.47	2.16	4.90
	**min**	1.26	0.13	2.41	0.19	0.00	0.19	0.87	0.31	**1.78**	**4.33**
	**max**	2.07	0.22	3.64	0.35	0.04	0.27	1.53	0.57	**2.66**	**5.92**
	**average**	1.70	0.17	3.02	0.26	0.02	0.21	1.22	0.46	**2.18**	**4.90**
	**SD**	0.29	0.04	0.50	0.05	0.01	0.03	0.24	0.09	**0.31**	**0.63**
